# Increasing advance personal planning: the need for action at the community level

**DOI:** 10.1186/s12889-018-5523-5

**Published:** 2018-05-09

**Authors:** Amy Waller, Rob Sanson-Fisher, Nola Ries, Jamie Bryant

**Affiliations:** 1 Health Behaviour Research Collaborative, School of Medicine and Public Health, Faculty of Health, University of Newcastle, Callaghan, NSW Australia; 2 Priority Research Centre for Health Behaviour, University of Newcastle, Callaghan, NSW Australia; 3 Hunter Medical Research Institute, New Lambton Heights, NSW Australia; 4 Faculty of Law, University of Technology Sydney, Ultimo, NSW Australia

**Keywords:** Advance personal planning, Capacity, Community action, End-of-life, Advance care planning

## Abstract

**Background:**

Advance personal planning is the process by which people consider, document and communicate their preferences for personal, financial and health matters in case they lose the ability to make decisions or express their wishes in the future.

**Discussion:**

Advance personal planning is most often undertaken by individuals who are seriously ill, often in the context of a medical crisis and/or at the time of admission to hospital. However, the clinical utility and legal validity of the planning process may be compromised in these circumstances. Patients may lack sufficient capacity to meaningfully engage in advance personal planning; there may be insufficient time to adequately reflect on and discuss wishes with key others; and there may also be limited opportunity for inter-professional input and collaboration in the process. Here, we propose an agenda for research to advance the science of advance personal planning by promoting a ‘whole community’ approach. Adoption of advance personal planning at a community level may be achieved using a variety of strategies including public media campaigns, intervening with professionals across a range of health care and legal settings, and mobilising support from influential groups and local government. One potentially promising method for encouraging earlier adoption of advance personal planning among a broader population involves a community action approach, whereby multiple evidence-based strategies are integrated across multiple access points. Community action involves calling on community members, professionals, community and/or government organisations to work collaboratively to design and systematically implement intervention strategies with the aim of bringing about desired behaviour change. An example of a community action trial to improving uptake and quality of advance personal planning is described.

**Conclusion:**

While promising, there is a need for rigorous evidence to demonstrate whether a community action approach is effective in establishing whole community adoption of advance personal planning.

## Background

### Advance personal planning (APP) is important, yet rates of participation are variable

Advance Personal Planning (APP) is a process of planning for future incapacity and the end of life. It can include: making an advance care directive (ACD) to express values and instructions about health matters; the appointment of substitute decision makers (SDMs) to make financial, health and personal decisions during periods of incapacity; and making a will to distribute assets upon death. Documented benefits to engaging in APP include reducing the likelihood of: estate distribution disputes, financial exploitation, unwanted medical care, and easing the burden on SDMs [1–3]. APP can also increase awareness and knowledge without increasing hopelessness or anxiety [4].

Despite the potential benefits, rates of uptake of APP vary. Many people do not engage in the full range of APP actions. People are more likely to complete financial APP instruments such as wills, than instruments that cover personal and health issues [2]. Studies on the prevalence of ACD ownership have been primarily undertaken in the USA, with figures ranging from 26 to 70% among those aged 65 years or over [5]. A 2014 survey of 15,617 older adults found that completion of written plans describing treatment options varied across 11 countries, including Germany (58%), Canada (46%), Australia (31%), the UK (20%), and Norway (4%) [6]. Despite their potentially central role in decision-making in cases where the patient does not have capacity and no valid ACD is available, the appointment of medical SDMs also occurs infrequently [6, 7].

### There are a number of disadvantages to absent or delayed APP

To engage meaningfully in APP, people need realistic and accurate information about choices and likely outcomes. However, these conversations are often delayed until people are imminently dying [8]. Consideration of who people want to make decisions and their most important values may be unrealistic in circumstances where conversations are delayed until a medical crisis and a person’s capacity is lost due to treatment or illness [8, 9]. There may be reluctance to initiate APP with people who are acutely unwell for fear that decisions may not accurately reflect their wishes, or that it will cause distress. While declining personal health is a strong motivator for APP, many people express a desire for discussions to occur sooner rather than later [10]. Those who are not seriously ill or have limited access to services may also miss opportunities for APP. This is an important gap as these individuals can still be called on to act as a SDM for someone else, or experience unexpected traumatic events that lead to a loss of their decision-making capacity. This places them at greater risk of negative outcomes, such as unintended distribution of assets, complications over guardianship of children or receipt of unwanted medical care [11].

Providers may also be reluctant to implement instruments established some time ago or that include instructions that are vague or do not comply with quality criteria set out in policy documents [12, 13]. Health-legal collaboration has been highlighted as one method for improving the quality of APP processes, particularly the legal validity and clinical utility of the documents that are prepared [14]. However, opportunities for input and collaboration across health and legal professions is often limited in health care settings or during medical crises. Working within professional siloes, neither lawyers nor doctors are optimally effective in helping clients with APP [15]. While their involvement may enhance clinical utility, health professionals are often challenged by limited legal knowledge in relation to APP [16]; and difficulties identifying those who lack capacity to make decisions [17]. Lawyers have inconsistent practices for assessing capacity [18] and generally lack awareness of the realities of healthcare delivery [15]. Competing provider demands and problems in sharing instruments with relevant stakeholders also contribute to the failure to successfully implement APP [19].

## Main text

### Reaching a broader population in a more timely manner: A whole community approach

Effective APP requires that a person is given time to consider and express values and preferences; someone is appointed who is able to make decisions on behalf of the person if required (i.e. an SDM); and documentation of values and preferences is available when decisions need to be made [20, 21]. A range of different approaches have been tested to improve the uptake and quality of APP. Traditionally, intervention strategies aimed at improving participation have been targeted toward defined individuals, groups or care settings (e.g seriouslly ill people, hospitals). Interventions providing standardised information to individuals have improved documentation of EOL preferences by 25–30% [22]. Combined interventions targeted toward patients and professionals, such as skills-based training, tailored pre-visit educational coaching interventions and question prompt lists, have also been effective in improving EOL communication [23]. Attempts to reach a broader spectrum of the population have led to intervention efforts that have been coordinated across multiple health care settings. For instance, system-level initiatives such as Respecting Patient Choices (RPC) and Physician Orders for Life-Sustaining Treatment (POLST), have used a combination of strategies, such as professional education, organizational policies and procedures and quality improvement [24]. These initiatives have achieved modest improvements in rates of ACD ownership, identification of appropriate SDMs and documentation of wishes [25, 26].

While promising, these initiatives have focused solely on improving engagement in medical decision-making. The range of complex decisions people may face as they transition through different life-stages (i.e. personal and financial as well as health) are rarely considered. Further, they do not allow for fluctuations that may occur in a person’s capacity, which is typically temporal and situational. For instance, a person may be capable of making simple health decisions (e.g. have a blood test), but incapable of making complex financial decisions (e.g. selling a house). Optimal APP therefore requires ongoing discussions of peoples wishes across multiple decision-making domains. Because the consequences of suboptimal APP may be spread across multiple settings, including health, legal and social services, all members of a community have a responsibility to work together to improve the quality of APP processes, rather than relying on efforts within the health care sector. Further, key APP decisions can cover multiple domains (e.g. financial implications of a decision about accessing services) which may require different professional skill sets. In response, there have been calls to “transform systemic processes across institutional settings” by involving a range of professionals, civic leaders, governmental and non-governmental organisations and community groups in promoting APP [27]. In essence, this describes a community action approach, but little work has been done to conceptualise the elements.

### A ‘community action’ approach to promote adoption of APP in the wider community

Community action involves implementing multiple intervention strategies across settings with community participation integral to implementation and maintenance [28]. Community stakeholders collaborate through a coalition to create and implement tailored strategic plans with demonstrated effectiveness in achieving desired change [28]. It is designed to build capacity within communities so members possess the requisite resources and skills to implement intervention strategies themselves [28]. Community action approaches been tested for a range of other health and lifestyle behaviours, including substance-abuse, cardiovascular disease, HIV, physical activity and cancer prevention [28–30]. Equivocal findings in addressing defined health and social problems have been reported, with a systematic review of community-based programs highlighting that many had only a modest impact [30]. This is in contrast to the success of this approach when applied to HIV prevention. While acknowledging the potential influence of the nature of the disease and its significant risks, the authors attribute this disparity in part to the poor methodological quality of the former studies [30]. In particular, the use of non-comparative designs; inadequate measures and analytic techniques; potential variation in the extent to which communities are exposed to different strategies; and insufficient follow-up time have prohibited any definitive conclusions being made about the effectiveness of community action approaches [30, 31]. Furthermore, community HIV interventions appear to have benefited from extensive formative research and a greater emphasis on changing social norms. Specifically, programs focused on role modelling, developing a sense of mastery in engaging in risk-reducing behaviours, and reinforcing educational messages [30]. For instance, using peer volunteers to deliver the health messages and tailored interventions and messages to reach various population subgroups rather than just targeting individuals [30]. More broadly, O’Mara and colleagues recommend the following strategies when implementing “community-engagement” interventions: involving community members in the delivery as well as the design of interventions; implementing universal as well as targeted interventions; incorporating active strategies, rather than just relying on passive information; and involving stakeholders outside of health [31]. Finally, a recent Cochrane review of “coalition-driven” interventions suggest that strategies which target health and legal professionals, change policies and procedures, and/or increase system capacity and infrastructure are able to produce small yet consistent results [32]. Providing education and support for at-risk groups via trained peers or professionals has produced consistent, moderate benefits [32].

### Well-controlled experimental research needed to test a community action approach APP

The effectiveness and cost-effectiveness of any intervention must be evaluated to maximize health and socio-economic benefits, minimize harms, avoid exacerbation of health inequities and maximize the value of resources [33]. To enable a thorough examination of impact and ensure that the findings produce high quality evidence suitable for translation, trials should include: a robust design to minimise contamination and bias; significant involvement of end-users to ensure responsiveness to consumer needs and relevance to policy and practice; ‘tailoring’ of low-burden, evidence-based strategies that can be implemented across multiple settings; robust outcome and process measures and statistical analysis [33, 34]. Allowing sufficient time for interventions to have an effect has also been highlighted [34]. The following information provides an example of how a community action approach to APP could be implemented and evaluated.

#### Step 1: Selection of communities

Selection should be guided by specific criteria including that they are: (a) defined in a way that those to whom the results are generalisable can be clearly delineated; (b) large enough to have sufficient resources to implement multiple interventions and small enough to allow observation of effects; (c) geographically distant to minimise the risk of contamination, enhancing methodological rigour; (d) representative of the general population to increase the likelihood that this model can be adopted by other communities; and (iv) potential confounding factors can be minimised [34, 35]. For APP, limiting to a single jurisdiction can avoid confounding based on legislative or structural differences between states.

#### Step 2: Holding a community forum in each intervention community

An initial approach is made to local government and council representatives by the research team to outline the purpose and nature of the study, and invite a representative to chair the forum. Invitations are sent to other stakeholder groups, including legal firms, general practices, aged care services, hospitals, community organisations, local media, and representatives from government departments with administrative or regulatory oversight [35]. Forums are also advertised via social and online media, as well as via local media and community organisations. Information about the purpose and nature of the planned intervention is presented and the community’s participation requested. Forum attendees are invited to form local Community-based  Coalition Groups.

#### Step 3: Community-based Coalition Groups are established

Community-based Coalition Groups are established to select, manage and enact intervention strategies. Membership will be dependent on local context, however should include individuals with legitimacy in the community. A first meeting should be held to decide on: roles and responsibilities; format of meetings; barriers and enablers to APP in the community; and the intervention strategies to implement. Standard resource packages can be developed to facilitate implementation and regular support is provided by the team via site visits and videoconference.

#### Step 4: Coalition group to select and implement intervention strategies

Each Coalition Group is given access to a range of strategies, included based on their feasibility, acceptability, and likelihood of achieving sustainable and meaningful change. The research team provides advice and support to communities to identify and prioritise specific barriers and enablers to APP, and select and implement strategies. The final decision about which strategies to implement rests with the Coalition Group. Some examples of strategies that might be employed include:

##### Strategy 1: Inter-professional education workshops to promote APP

Inter-professional education in medico-legal issues and communication skills can help address common barriers to APP. For instance, a lack of confidence in capacity assessment and end of life planning among health professionals [16]; and a lack of awareness of the practical realities of healthcare delivery among lawyers [15]. Directed by two facilitators (e.g. a lawyer & health professional from the community), a combination of proactive, practice-oriented strategies (e.g. role-plays), group discussions and feedback can be used to optimise communication skills. Topics might include: benefits of APP; the legal framework, including capacity; and roles and responsibilities of patients, SDMs, lawyers and healthcare providers. Access to a suite of resources can be made available to attendees. For instance, links to existing capacity assessment toolkits; downloadable APP templates and legal health checklists; as well as training modules with self-assessments to demonstrate and reinforce key issues.

##### Strategy 2: Community APP sessions for older people led by health and legal practitioners

Currently, the quality and nature of the information people receive about APP is likely to vary according to the skills, time, knowledge and personal preferences of the person giving information [8, 16, 36, 55]. Participatory approaches that raise awareness and encourage people to engage in APP while they still have capacity are required. Members of volunteer and community organisations and clients of aged care facilities can be targeted for participation. Eligible organisations are informed about the free APP information session(s) and asked to invite members. Sessions are advertised via local media and community-wide mailouts. Facilitated by trained health and legal professionals, information on the purpose and benefit of APP; legal rights and responsibilities; and techniques for facilitating discussions are presented. Previous studies also highlight the potential benefit of adopting a dyadic approach to APP. Greater emotional support, better relationship quality and having been hospitalised or undergone surgery (for men) has been positively associated with planning; while poor spousal health (among wives) is negatively associated with ACD completion [37, 38]. These studies recommend joint planning sessions involving patients and spouses, with strategies to facilitate discussions between spouses about how to manage the potential impact of poor spousal health on decision-making [38].

##### Strategy 3: Information for people attending health and legal services regarding APP

Provision of information by professionals (e.g. GPs, lawyers) increases the likelihood that people will adhere to recommended behaviour changes. These professionals are also frequently accessed by the general population. For instance, GPs have access to a high proportion of the population [39]. People are also increasingly seeking legal help, including via telephone advice and advocacy services (83% increase), with wills, powers of attorney, and guardianship issues being the most common problems [40]. Providing information in waiting rooms offers a practical and time-efficient method for promoting APP. Coalition Groups can approach health services and legal firms for consent to place print materials including printed posters in waiting rooms. Information about the potential benefits of APP, local services and additional resources available to assist people to engage can be provided.

##### Strategy 4: Opportunistic assessment and feedback in health and legal services

Variation in the type and quality of information gathering and transfer can occur as a consequence of time and resource constraints, as well as individuals’ bias. Self-report assessments can be used to improve data integrity by standardising information collected on APP (e.g. willingness to prepare instruments, prior participation) via cross-sectional samples of clients. Data can be measured continuously and responses fed back to professionals in real-time to enable them to encourage or act on clients’ expressed wishes about engaging in APP. Items can also be integrated with questions about other health and lifestyle behaviours; with self-report data confirmed by provider reports (e.g. *Step 6*). Waiting-room screening and data collection is feasible and acceptable across a broad range of health care settings, including general practices, hospitals and Aboriginal Medical Services [41–44]. GPs for example are well-placed to integrate this strategy, as the majority of people visit at least once a year, they often have trusted relationships with patients and people are more likely to have the capacity to engage in discussions, when compared to many hospitalised patients or residents of aged care facilities. Completion of surveys is not disruptive to clinic functioning and can be easily incorporated into usual practice [41].

##### Strategy 5: Public awareness campaigns

Community awareness of the purpose and benefits of APP is limited [45]. Media campaigns can influence social and health behaviours across large populations, by providing focused messages to large audiences repeatedly, over time, and at a low cost per head [46]. Media that uses local data to generate news items may be more effective than relying solely on public service announcements [34]. The purpose of this strategy is to generate a substantive increase in the number of APP-related news items. The benefits of APP and services available to assist in APP preparation can be communicated by: regular advertisements in local newspaper/radio; media releases; short videos played as part of loop advertising at community organisations (e.g. bowling clubs, RSLs); written leaflets distributed via mail outs (e.g. with council rate notices); posters and handouts distributed in public libraries and/or pharmacies.

##### Step 6: Performance feedback to monitor APP over time

Legal and health professionals (e.g. GPs) in each intervention community can collect data on the number of clients who request, complete or update APP instruments during the intervention period. Data is collected every two months to ensure accuracy, with follow up reminders to enhance adherence. Community-specific data is mapped against previous data to monitor progress and presented to Coalition Groups at ‘feedback sessions’ periodically to aid monitoring of strategies. As there is likely to be variation across communities in rate of improvement, a stepped approach involving a transition from lesser to more intensive strategies may be employed if improvements seen after a defined period [47].

### Evaluation of impact community action on uptake and quality of APP

A recent Delphi study highlighted the lack of consensus regarding the most important outcomes that define successful implementation of end of life planning, making it difficult to compare findings across initiatives [48]. This group recommend a broad set of outcomes to evaluate planning initiatives across four domains, including: actions, process, quality of care and health care [49].

#### Action outcomes

In some countries, such as Australia, no standardised data or formal documentation of uptake currently exists, which can also limit attempts to evaluate the impact of interventions [48, 50]. However, new initiatives for establishing the national prevalence of ACP via audits and self-report surveys are currently being tested [50]. If successful, this may offer an important source of baseline data for trials of APP initiatives, including community action approaches [50]. In the meantime, measures of APP uptake will need to rely on self-report surveys of random samples of people residing in each community at multiple time points. The proportion of APP instruments made (or updated) can be collected and compared across communities. Information on the quality of APP processes, including timing, involvement of others and whether these instruments have been shared with key stakeholders can also be compared. Self-report data can be complemented by routinely collected audit data from health and legal services where available.

#### Process outcomes

These include outcomes such as individual’s behaviour, beliefs and attitudes [49]. Measures such as the *Advance Care Planning Engagement Survey* can be included to assess behaviour change domains such as APP knowledge, contemplation, self-efficacy, and readiness [51]. Perceptions of barriers and enablers to APP can be collected via self-report surveys of community members. Surveys of lawyers, health professionals and service managers within target communities can be undertaken to obtain their views about provider- and system-related barriers and enablers to APP and inter-professional collaboration across multiple sectors, including general practice, aged care and acute care.

#### Quality of care outcomes

Evidence that increased adoption of APP translates to improved end-of-life outcomes, such as concordance between preferred and actual care, higher quality of death and reduced family distress can be difficult to achieve [3]. For instance, there are no validated or ‘gold-standard’ procedures for measuring concordance between preferred and actual care. Real-time assessments are recommended, given changes in preferences and circumstances are likely to occur [49]. Bereavement follow-up undertaken with support persons of those who died under the care of hospitals, residential aged care facilities or palliative care services in target communities may provide an opportunity to explore concordance and quality of care received at the end of life.

Data sources such as applications to guardianship tribunal that involve disputes over medical decision-making for person who lacks capacity and complaints made to hospitals or to bodies like Healthcare Complaints Commissions regarding end of life care issues may also be sought [52].

#### Health care outcomes

Costs for additional resources required by each intervention strategy, such as time and materials required to deliver the strategies can be estimated; as well as the resources required to translate the intervention strategies to other communities. The cost of delivering community action can be compared to the consequence, defined by completion of APP instruments.

#### Exposure to intervention strategies

Exposure to intervention strategies should also be collected from intervention community members, health and legal professionals. These include the: (i) number of Coalition Group meetings held and attendance; (ii) number of inter-professional workshops held, number of attendees and attendee-reported content acceptability and utility; (iii) number of community information sessions held, number of attendees and attendee-reported content acceptability and utility; (iv) number of health and legal services providing information in waiting rooms; (iv) proportion of eligible clients screened in legal and health services and number of APP requests; and (v) number of APP-related articles in local media.

### Potential advantages and disadvantages of a community action approach to APP

Despite increasing interest in the potential for a community action approach to APP, there has been no rigorous evaluation. This makes it difficult to weigh its value relative to other strategies, such as legislative approaches and individual-directed interventions. There are a number of challenges associated with community action approaches. The multicomponent intervention is likely to have synergistic effects, so it may not be possible to delineate individual contributions of each component in achieving outcomes. Communities may also vary in the level of resources they have available to implement interventions, such as rates of GPs and/or lawyers per capita [34]. This will likely influence how effectively services implement strategies. Efforts should focus on strategies that have the potential to be embedded in routine practice with minimal resources. While there are likely to be difficulties in implementing and evaluating such a large and diffuse intervention, frameworks such as the Medical Research Council’s ‘Framework for design and evaluation of complex interventions to improve health’ can be applied to facilitate and support implementation. However, the first task is to demonstrate that treatment effects can be achieved with community action.

If effective, future research can examine the contribution of individual strategies. Further, national consistency in laws may be desirable to support APP in countries, like Australia, Canada and the USA, where states and provinces have varying laws for advance planning [7]. Legislative variation can cause confusion and uncertainty among healthcare providers which, in turn, is a barrier to engaging people in APP [7, 16]. Sustained improvements in APP practice might also be enhanced if it is linked to policies such as provider reimbursement or accreditation. In 2016, Medicare started reimbursing clinicians for ACP in the USA [53]. In Australia, requirements to have systems for preparing and documenting ACP in the clinical record are included in new mandatory accreditation standards [54]. The impact of these policies is yet to be determined.

Despite these challenges, there are a number of potential advantages of community action in the context of APP. Firstly, it affords communities the opportunity to systematically select, implement and evaluate a menu of effective behaviour change strategies concurrently across multiple sectors of the community [34, 35]. This is particularly promising for APP, given the increasing awareness of the need for health and legal professionals to work together as important sources of APP advice and support for clients [14]. Community action therefore has the potential to bridge service provision to meet peoples’ social, economic and health needs [14]. Offering choice to communities about the strategies they employ can also increase the likelihood of adherence, generalisability and representativeness by encouraging widespread buy-in [34, 35].

Secondly, it demonstrates principles of equity and access as strategies that promote APP to the broad community can be complemented by those targeting underserved groups [28]. For instance, given GPs’ significant population reach and often longstanding, trusted relationships with clients, strategies such as education and training and opportunistic screening can be used to support GPs to routinely encourage APP [55]. In contrast, providing training and resources to Indigenous Health workers in Aboriginal Medical Services might be more effective for promoting APP within this population, as they may be more likely to seek support from this trusted source [56]. Strategies can be implemented simultaneously across settings to enhance effectiveness for the wider community.

Thirdly, utilising multiple strategies that can reach a broader audience and are supported within existing community resources may be more cost-effective and sustainable than previous initiatives. For example, public media campaigns can be reach large audiences repeatedly and at a low cost [46], so may be more likely to be sustained. They can also raise awareness among groups who may not typically be exposed to APP conversations (e.g. younger people). This may prompt earlier conversations and more opportunity to revise choices if/when circumstances change. This can reduce the likelihood of urgent decisions and poorer outcomes [55].

## Conclusions

APP is typically delayed until people are imminently dying or during hospital admissions or emergency department visits. However there is increasing recognition that community-wide adoption among those who are otherwise well may have beneficial outcomes without significant adverse effects. Wide uptake of APP has been limited by a failure to ensure adequate mechanisms are in place to allow for a wider cross-section of the community to participate. Previous efforts to improve uptake have focused primarily on increasing the rate at which individuals plan ahead for medical decisions. Often these initiatives have adopted a ‘siloed’ approach, in which initiatives to promote engagement are directed towards patients or professionals in isolation without addressing larger systemic challenges. The proposed community action approach has the potential to reach a broader population in a more timely manner by integrating multiple evidence-based strategies and mobilising support from a range of influential groups. While promising, there is a need for rigorous evidence about whether benefits of this approach outweighs the costs.

## Abbreviations

ACD: Advance care directive
APP: Advance personal planning
POLST: Physician Orders for Life-Sustaining Treatment
RPC: Respecting Patient Choices
SDM: substitute decision-maker
UK: United Kingdom
USA: United States of America
